# A pilot study testing the feasibility of skin temperature monitoring to reduce recurrent foot ulcers in patients with diabetes – a randomized controlled trial

**DOI:** 10.1186/s12902-015-0054-x

**Published:** 2015-10-09

**Authors:** Anita Skafjeld, Marjolein. M. Iversen, Ingar Holme, Lis Ribu, Kjetil Hvaal, Bente. K. Kilhovd

**Affiliations:** Department of Endocrinology, Morbid Obesity and Preventive medicine, Oslo University Hospital, Oslo, Norway; Department of Health and Social Sciences, Bergen University College, Bergen, Norway; Department of Medicine, Section of Endocrinology, Stavanger University Hospital, Stavanger, Norway; School of Sport Sciences in Oslo, Oslo, Norway; Faculty of Health Sciences, Oslo and Akershus University College of Applied Sciences, Oslo, Norway; Department of Orthopedic Surgery, Oslo University Hospital, Oslo, Norway

**Keywords:** Diabetic foot, Foot ulcer, Thermometry, Motivation, Preventive health services

## Abstract

**Background:**

Although monitoring foot skin temperatures has been associated with diabetic foot ulcer recurrence, no studies have been carried out to test the feasibility among European Caucasians. Moreover, the educational and/or motivational models that promote cognitive or psychosocial processes in these studies are lacking. Thus, we conducted a pilot randomized controlled trial to test the feasibility of monitoring foot skin temperatures in combination with theory-based counselling to standard foot care to reduce diabetic foot ulcer recurrence.

**Methods:**

In a single-blinded nurse-led 1-year controlled trial, conducted at a hospital setting in Norway, 41 patients with diabetic neuropathy and previous foot ulcer were randomized to the intervention (*n* = 21) or control groups (*n* = 20). All participants were instructed in foot care and recording observations daily. Additionally, the intervention group was taught how to monitor and record skin temperature at baseline, and received counselling every third month supporting them to use the new treatment. Subjects observing temperature differences >2.0 °C between corresponding sites on the left and right foot on two consecutive days were asked to contact the study nurse and reduce physical activity. Fisher exact test was used to evaluate the effect of the intervention on the proportion of subjects with a foot ulcer. Kaplan-Meier survival analysis was performed to compare the two groups in regard to the time to development of a foot ulcer.

**Results:**

In the intervention group, 67 % (*n* = 14/21) monitored and recorded skin temperatures ≥80 % of the time while 70 % (*n* = 14/20) of the controls recorded foot inspections. Foot ulcer incidence was 39 % (7/21) vs. 50 % (10/20) in the intervention and control groups, respectively (ns).

**Conclusions:**

This feasibility study showed that the addition of counselling to promote self-monitoring of skin temperature to standard care to prevent recurrence of foot ulcer is feasible in patients with diabetes in Norway. Home skin temperature monitoring was performed as frequently by the intervention group as usual foot observations in the controls despite the extra effort required. We did not detect a difference in foot ulcer recurrence between groups, but our study may inform future full scale studies.

**Trial registration:**

Clinicaltrials.gov NCT01269502

## Background

Foot ulcers are a feared complication of diabetes. Rates of diabetes-related complications have declined substantially in the past two decades, but given the increasing prevalence of diabetes, foot ulcers may be expected to increase worldwide [1–3]. After a first foot ulcer, the risk of recurrence is as high as 30–87 % [4, 5]. One possible reason for the high recurrence rates is that sensory neuropathy reduces warning symptoms of pain and inflammation, the major early signs of skin damage and ulceration [6, 7]. Thus, eliciting objective signs of early damage may be useful. Several interventions to reduce the frequency of diabetic foot ulcers have been tested [6, 8–10]. Three of these studies were randomized controlled trials testing patient monitoring of foot skin temperature as a warning signal of an impending ulcer [6, 9, 10]. These studies found a significant reduction in new foot ulcers with use of a temperature monitoring device. Although monitoring foot skin temperatures has been associated with diabetic foot ulcer recurrence, no studies have been carried out to test the feasibility in Norway and/or among European Caucasians.

Another important aspect of foot ulcer prevention is patient education. A systematic review found that most studies failed to address the educational and/or motivational models that promote cognitive or psychosocial processes [8]. Ongoing self-management education and support are important to prevent diabetes complications [11], and individuals’ readiness to change behavior may improve the outcome. An individual’s preparedness can be divided into a pre-action phase or into an action-phase according to motivation, and in the Transtheoretical Model (TTM) this may be classified into five stages: pre-contemplation, contemplation, preparation, action, and maintenance [12]. Counselling may be tailored toward the stage of change. Use of this model has shown positive results in a number of studies that aimed to facilitate lifestyle and behavior change [13, 14].

Thus, we conducted a pilot randomized controlled trial to test the feasibility of monitoring foot temperature in combination with theory-based counselling with the ultimate aim of preventing recurrent diabetic foot ulcers. The study was conducted at a diabetes specialty clinic in a university hospital in Norway.

## Methods

The study was a single blind randomized controlled pilot trial with a 1-year follow-up period. Block randomization was used to assign each four subjects to blocks with two in each group. Randomization was stratified for patients with a history of Charcot foot, who have an extra high risk of recurrence. All study related procedures including randomization were performed at the Diabetes Clinic, Oslo University Hospital.

The study was reviewed by the Regional Committee for Medical and Health Research Ethics South East (2009/1129). The participants were guaranteed full confidentiality and each participant gave written informed consent.

**Fig. 1:**
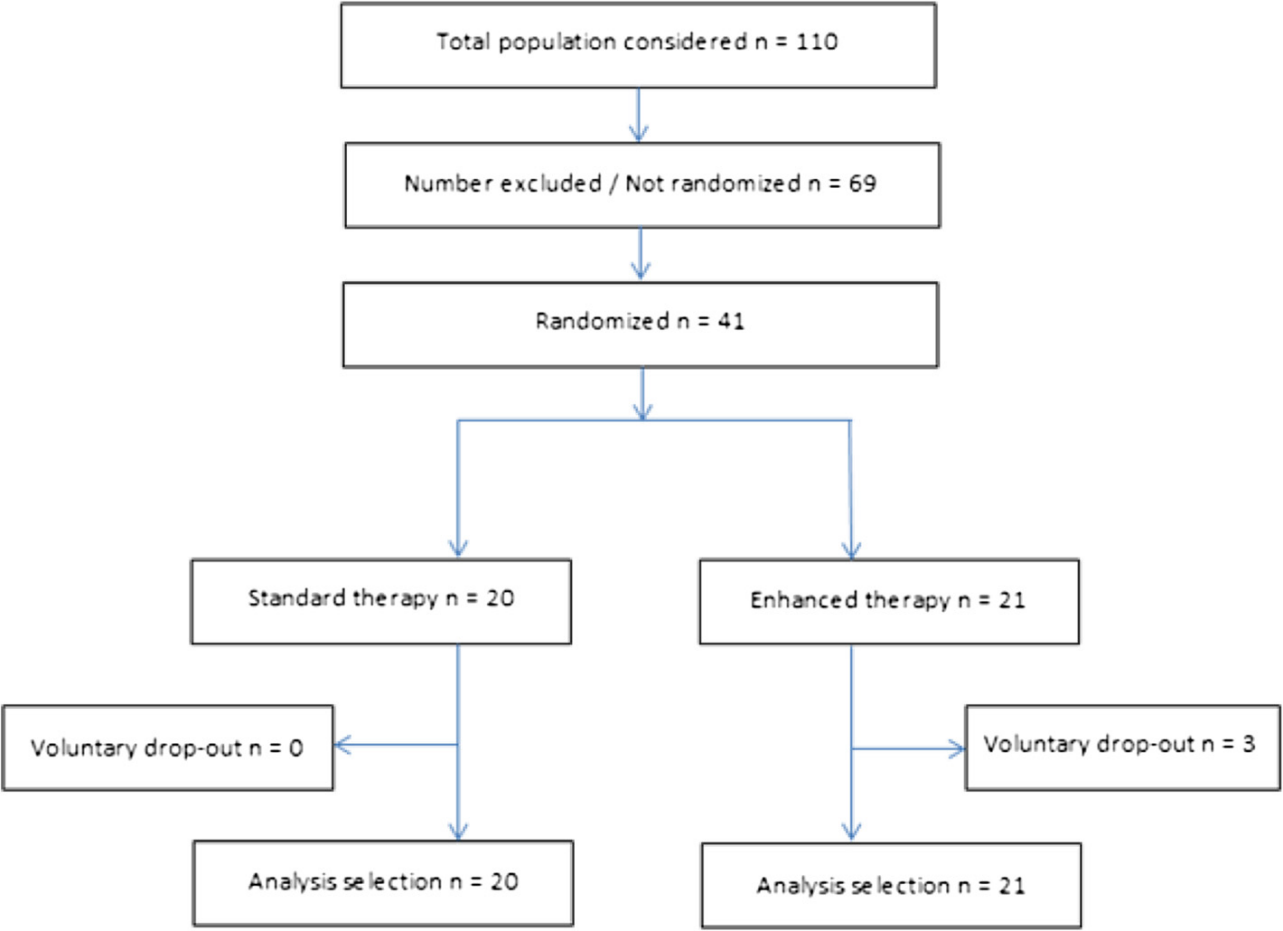
Study enrolment

Participants were recruited from six diabetes specialty outpatient clinics and one chiropodist in the Oslo area. Inclusion criteria were a diagnosis of type 1 or type 2 diabetes, age 18–80 years, and belonging to group 3 of the Diabetes Foot Risk classification system (previous history of foot ulcer and peripheral neuropathy) [15]. Study participants had to be capable of providing informed consent and completing a written questionnaire. Study participants also had to be willing to measure foot skin temperature if assigned to the intervention group. Exclusion criteria included open wounds, active Charcot disease, active osteomyelitis, or ischemia (not palpable pulses or ankle/arm index <0.7). Of 110 patients screened, 41 patients fulfilled the study criteria, provided consent and were randomly assigned to the intervention (*n* = 21) or control group (*n* = 20) (Fig. 1). All patients were Caucasian.

The control group was instructed to inspect their feet under, below and between the toes, and record their observations in a log book daily. They were instructed to contact the study nurse if changes in their feet including a new ulcer were observed. They were also advised to always wear their customized footwear. For general medical and diabetes care, they consulted their usual general practitioners.

In addition to the same standard care as the control group, the intervention group was trained to use a digital infrared thermometer (Temp Touch; Xilas Medical, San Antonio, TX) to monitor foot temperature. The thermometer is a handheld device with an infrared heat sensor. Study subjects were instructed to record daily physical activity using a step-counter (Yamax, Tokyo, Japan) during the first week of the study.

At the baseline visit, the study nurse explained the purpose of the thermometer and how to conduct daily self-monitoring of skin temperatures. Temperature was to be monitored at the same six points under both feet and recorded in a log book daily. If subjects observed a difference in skin temperature of >2.0 ° C (2.2 ° C = 4 ° F) on the same spot in the sole of the foot compared to the same spot on the opposite foot on two consecutive days, they were advised to contact the study nurse and to reduce physical activity by one-half until the temperature difference normalized (to <2.0 ° C).

At three-month visits, the study nurse assessed each subject’s readiness to record skin temperatures according to TTM stages, followed by tailored stage-based counselling. The five stages were modified as follows: 1) “I am not using the thermometer, and do not intend to use it”, 2) “I am thinking of using it, but not in the very near future”, 3) “I will start to use it now”, 4) “I have started to use it, but not regularly (<80 % of the prescribed time)”, 5) “I am using it regularly (≥80 % of the prescribed time)".

At baseline and at the end of the study, participants completed questionnaires that included socio-demographic variables (age, gender, living conditions, education and employment status), lifestyle (smoking habits), diabetes-related variables (diabetes type and duration, co-morbidities, history of foot ulcers, Charcot foot and any history of lower limb amputation) and patient related outcomes. At the end of the study the participants additionally completed a questionnaire about how often they used customized footwear.

Clinical examinations were performed at baseline and at study-end. The study nurse measured waist circumference, body weight and height (baseline visit only) to calculate body mass index (BMI), and conducted an extensive foot examination including assessment of pedal pulses and ankle-arm index, bone, trophic changes in the skin and nails of the feet, and sensory monofilament and vibration tests. The monofilament test results were categorized as reduced (7 out of 8 or less) or absent sensation. The vibration perception threshold was categorized as able to sense the vibration or unable to sense the vibration. An orthopedic surgeon (KH) and an endocrinologist (BK) conducted clinical examinations of each participant at baseline and study end. Glycemic control was measured by HbA_1c_ [16]. Nephropathy was defined as urinary albumin/creatinine ratio (ACR) ≥3.0 [17, 18].

In the intervention group adherence to skin temperature monitoring was recorded as the percentage of days with foot temperature measurements recorded in the daily log in the course of the study. In the control group the percentage of days with a check indicating foot inspection was recorded in the daily log in the course of the study. Foot ulcer occurrence was defined as an end point in the study and was classified according to Wagner foot classification system.

For the purpose of analyses, monitoring of skin temperature was divided into categories of <80 % or ≥ 80 % of prescribed. Independent-sample *t*- tests were used to evaluate between group comparisons on continuous variables (age, BMI, waist circumstance, HbA_1c_, duration of diabetes and ankle-brachial index). Fisher exact test was used to evaluate the effect of the intervention on the proportion of subjects with a foot ulcer. Kaplan-Meier survival analysis was performed to compare the two groups in regard to the time to development of a foot ulcer. Differences between survival curves in the two groups were tested with the logrank test. Statistical analyses were undertaken using SPSS (PASW Statistics for Windows, Version 18.0. Chicago: SPSS Inc.). Statistical significance was assessed with two-sided *p* < 0.05. All analyses were by intention to treat.

## Results

There were no significant differences in demographics characteristics between the intervention and control groups at baseline. Nephropathy and systemic vascular risk factor levels above the recommended limits were more prevalent in the intervention than in the control group (Table 1). Only one subject in the study population reached recommended targets for all levels.

**Table 1:** Description of the study population

Characteristics	Control group	Intervention group
	*n* = 20^a^	*n* = 21^a^
Sociodemographic characteristics		
Age (years)	59,4 (SD 13.0)	57,1 (SD 10, 2)
Male sex (%)	75	86
Living alone (%)	40	33
Education (>12 years) (%)	40	33
Working (full/part time)	45	52
Lifestyle characteristics		
BMI (kg/m2)	31.1 (SD 6.4)	31.4 (SD 4.8)
Waist circumference (cm)	110.9 (SD 13.7)	114.9 (SD 13.1)
Current smokers (%)	30	19
Subgroups of diabetes		
Type 1 (%)	30	29
Type 2 (%)	70	71
Diabetes-specific variables		
HbA_1_ (mmol/mol)	63 (SD 19)	67 (SD 16)
HbA_1c_ (% units)	7.9 (SD 1.7)	8.3 (SD 1.5)
Insulin use (%)	55	71
Urinary albumin/creatinin ratio^b^	20*	65 *
Nephropathy (%)	25	48
Duration of diabetes (years) (median)	19.5	17.0
Vascular surgery/ PTA/blocking (%)	15	33
Retinopathy (%)	47	40
Foot surgery (%)	55	53
Charcot foot (%)	20	14
Multiple ulcer history (%)	85	65
A hx of previous toe amputation (%)	40	33
A hx of previous multiple ulcers (%)	85	62
Time since last healed ulcer		
<3 months (%)	40	52
3–6 months (%)	35	29
6–12 months (%)	15	9.5
>12 months (%)	10	9.5
Lower extremity examination		
Neuropathy evaluation		
Semmes-Weinstein 10-g monofilament right (*n*)^c^	15/5	13/8
Semmes-Weinstein 10-g monofilament left (*n*)^c^	18/2	12/9
Vibration perception threshold right (*n*)^d^	7/13	7/14
Vibration perception threshold left (*n*)^d^	9/11	9/12
Foot deformity		
Hallux valgus (*n*)	15	12
Claw toe (*n*)	26	20
Cavus (*n*)	10	19
Charcot (*n*)	4	5
Vascular examination		
Ankle-brachial index right	1.2 (SD 0.22)	1.2 (SD 0.25)
Ankle-brachial index left	1.2 (SD 0.25)	1.1 (SD 0.12)
Footwear compliance		
Time prescribed shoes were worn (hours)		
<4 (%)	10	16
4–8 (%)	25	28
>8–12 (%)	30	28
>12 (%)	35	28
The use of prescribed shoes		
Outdoor (%)	30	33
Indoor (%)	5	0
Both in- and outdoor (%)	60	61
Not used (%)	5	5.5
Risk factors (*n*)^e^	5/6/13/1	16/12/18/16

Data are means (SD) or %. ^a^Sample sizes vary somewhat depending on the actual completion of the different tests and questionnaires. ^b^Percentage with ACR >3. ^c^The results of monofilament test are divided into two categories: reduced sensation (7 out of 8 or less) or absent sensation. ^d^The vibration perception threshold was also divided into two categories: able to sense the vibration or unable to sense the vibration. ^e^Risk factors: HbA1c >7 %/LDL >2,5/BP >130/80/ACR > 3. *Significant difference *p* < 0,01

**Fig. 2:**
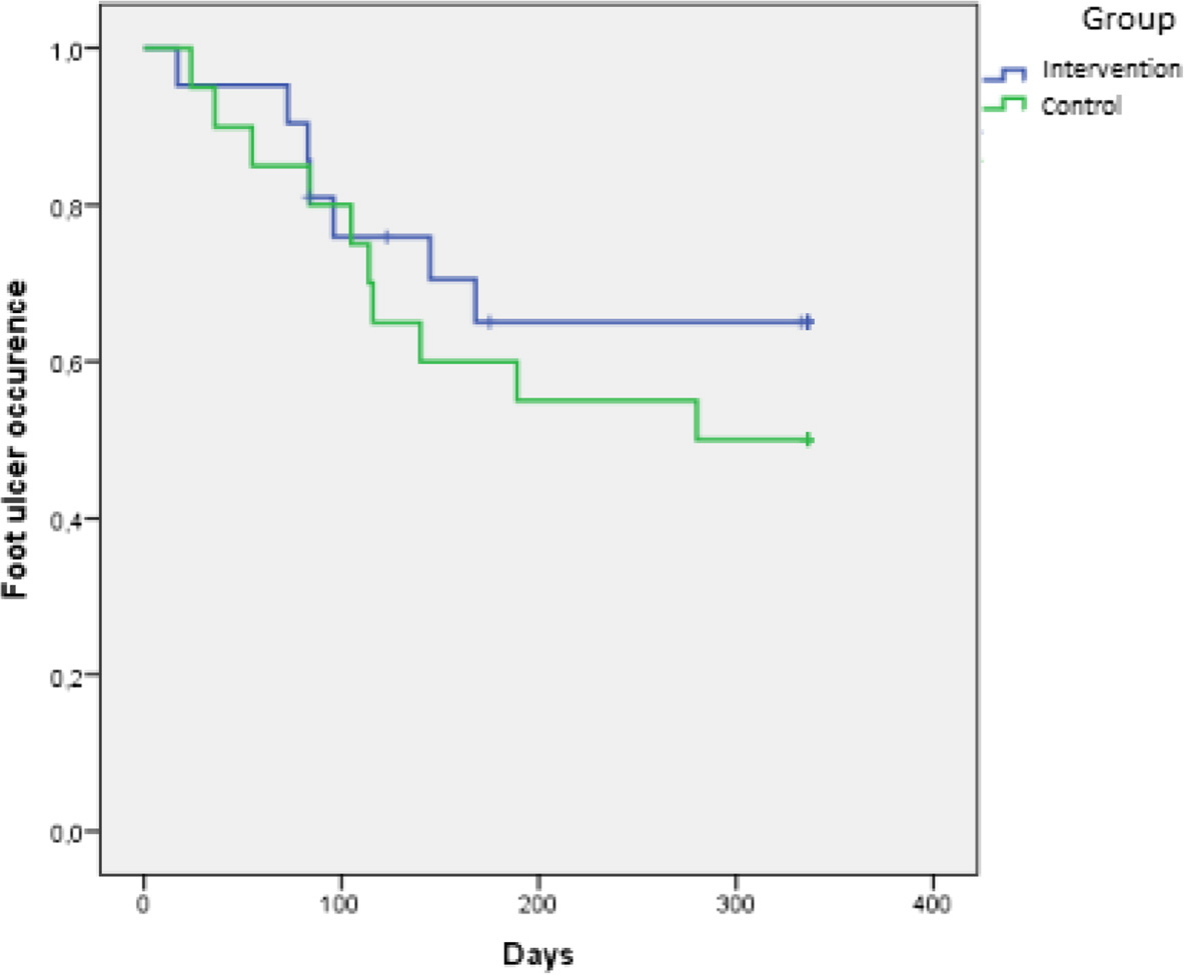
Kaplan-Meier survival curves comparing the time to develop a foot ulcer by treatment group. *P* = 0.407(Log Rank test)

In the intervention group, 67 % (14/21) recorded foot observations and skin temperatures ≥80 % of the time. In the control group, 70 % (14/20) recorded foot observations ≥80 % of the time. During the 1-year follow-up, the incidence of foot ulcers in the intervention and control group was 39 % (*n* = 7/21) and 50 % (*n* = 10/20) respectively (*p* = 0.532) (Table 2). Kaplan-Meier survival curves for the intervention and control group did not differ significantly (*p* = 0.407, chi-squared at 0.687) (Fig. 2).

**Table 2:** Clinical outcomes and voluntary withdrawals

	Control group	Intervention group
	(*n* = 20)	(*n* = 21)
Patients who contacted study nurse after self- examination		
Worried	4	4
Ulcer	8	6
Foot ulceration	10	7
Voluntary withdrawal from the study	0	3^a^

^a^Dropout (*n* = 1) and illness (*n* = 2)

In the intervention group there was no association between temperature monitoring ≥80 % compared to monitoring <80 % of the time to foot ulcer occurrence. In the intervention group 8 out of 21 experienced an increased skin temperature one or more times during the study. Fifty percent (4/8) of the patients from the intervention group and 33 % (4/12) of the patients from the control group contacted the study nurse, due to increased skin temperature and observed foot changes respectively, none of these developed a foot ulcer (Table 2).

Of the total sample, 24 % (*n* = 5/21) in the intervention group and 35 % (*n* = 7/20) in the control group used customized footwear >12 h a day (*p* = 0,858). During the study, there were no major changes in the feet of the participants until they had a recurrent foot ulcer (which was the predefined study endpoint) or they stayed in the study until the end-of-study visit at 1 year. In participants who contacted the study nurse due to worries about their feet, there were no major pathologies.

## Discussion

This pilot study is, to our knowledge, the first trial reported in a European setting using a skin temperature device and TTM model as a tool for health care personnel to prevent recurrence of diabetes related foot ulcers. Although we did not detect a difference in foot ulcer recurrence between groups, the study showed that the intervention is feasible in patients with diabetes and may inform future, adequately powered studies.

Intervention group patients in our study had a very high level of adherence with foot temperature recording. There was no difference between the intervention and control group regarding the frequency of contact with the study nurse. This is in contrast to results from the study performed by Lavery et al. [6] which showed that among patients in the intervention group who developed foot ulcers, 80 % did not comply with temperature assessment, and there were fewer study nurse contacts. The high level of adherence in the present study could be related to participants having to be initially motivated to perform temperature assessment and thus, there might be a selection bias. On the other hand, participants in both the intervention and control group identifying concerns with their feet with the same frequency might contribute to the lack of difference between the groups. The study nurse was easily available at day time. Future studies may need to emphasize the importance of contact with health personnel when temperature rises are noted.

The study by Lavery et al. [6] found an incidence of recurrent foot ulcer of only 8.5 % in the enhanced therapy group (temperature monitoring, taught by videotape and checked by study nurse), over a period of 15 months. We found higher recurrence rate of foot ulcers in the intervention group (39 %). One explanation of the difference might be that we included patients who had high levels of vascular risk factors with levels of HbA_1c_, LDL-cholesterol, blood pressure and urine albumin/creatinin ratio above recommended treatment targets. Significantly more patients in the intervention group had increased urinary albumin excretion (Table 1). Participants in our study could thus be more at risk for recurrence of foot ulcers [15]. Our findings underline the importance of starting monitoring foot skin temperatures of those at risk for foot ulcers at an early stage.

The use of customized footwear was substantially below recommendations in both groups. Footwear cannot be effective if it is not worn, and adherence is known to be low in diabetic patients [19]. A Randomized Controlled Trial among patients with foot pathology shows that the design and user friendliness of footwear is an important factor for compliance [20]. In self-management and support to patients with diabetes and high-risk foot conditions, more emphasize should be on the use of customized footwear indoors.

This study has limitations. The current study is underpowered to show differences between study groups. The sample size is too small to detect differences in moderate effect sizes between the intervention and the control group. Based on the pilot study results, 124 participants per group would be needed to have 80 % power to detect a difference at 5 % significance level. However, the aim was to test the feasibility of introducing temperature measurement in secondary prevention of diabetic foot ulcers in specialist centre in Norway. Another limitation in our study was that we implemented two interventions, the use of a thermometer for monitoring together with a theory-based counseling by a specialist diabetes nurse. This makes it difficult to separate the effect of each component and limits direct comparisons with previous studies. In our study the study nurse met the patients in the intervention group every third month. Effective communication with the patients is the cornerstone in a nurse-patient relationship, and vital to the providing of good care to the patient in hospital [21]. However, it takes time to build trust and motivation in order to change patient behavior and it is timely to raise the question if more frequent contact, perhaps monthly, or contact such as via telephone or email or Skype, could have strengthened the intervention. In spite of these limitations this feasibility study should provide knowledge to estimate important parameters that are needed to design the main study, (willingness of patients to be randomized, inclusion criteria, number of people eligible, follow-up rates, response rates and adherence/compliance rates) [22].

## Conclusions

With the aging of our population, the increased incidence and prevalence of diabetes, and the high personal and economic costs of diabetic foot ulcers, further attempts to improve clinical outcomes are merited. This feasibility study showed that the addition of counselling to promote self-monitoring of skin temperature to standard care to prevent recurrence of foot ulcer is feasible in patients with diabetes in Norway. Although we did not find any significant differences in foot ulcer development in the two groups, the knowledge gained in this study about the use of self-monitoring devices in combination with the use of theory-based counseling for patients with diabetes at risk for foot ulcers, may inform future full scale interventions to improve behavior change and foot ulcer recurrence.

## Abbreviations

ACR: Urinary albumin/creatinine ratio
BMI: Body mass index
HbA_1c_: Glycated hemoglobin A_1c_
TTM: Transtheoretical model
